# Role of FcγRIII in the nasal cavity of BALB/c mice in the primary amebic meningoencephalitis protection model

**DOI:** 10.1007/s00436-023-07810-w

**Published:** 2023-03-13

**Authors:** Diego Alexander Rojas-Ortega, Saúl Rojas-Hernández, María Elena Sánchez-Mendoza, Modesto Gómez-López, Jennifer Viridiana Sánchez-Camacho, Erika Rosales-Cruz, María Maricela Carrasco Yépez

**Affiliations:** 1 Laboratorio de Inmunología Molecular, Instituto Politécnico Nacional, Escuela Superior de Medicina, Salvador Diaz Mirón Esq. Plan de San Luis S/N, Miguel Hidalgo, Casco de Santo Tomas, Ciudad de Mexico, México; 2 Laboratorio de Investigación en Hematopatología, Departamento de Morfología, Escuela Nacional de Ciencias Biológicas, Ciudad de Mexico, México; 3 Grupo de Investigación CyMA, UIICSE, UNAM FES Iztacala, Los Reyes Iztacala, Tlalnepantla, Estado de México México

**Keywords:** *Naegleria fowleri*, Neutrophils, FcγRIII, IgG, Syk, Hck

## Abstract

Different mechanisms of the host immune response against the primary amebic meningoencephalitis (PAM) in the mouse protection model have been described. It has been proposed that antibodies opsonize *Naegleria fowleri* trophozoites; subsequently, the polymorphonuclear cells (PMNs) surround the trophozoites to avoid the infection. FcγRs activate signaling pathways of adapter proteins such as Syk and Hck on PMNs to promote different effector cell functions which are induced by the Fc portion of the antibody-antigen complexes. In this work, we analyzed the activation of PMNs, epithelial cells, and nasal passage cells via the expression of Syk and Hck genes. Our results showed an increment of the FcγRIII and IgG subclasses in the nasal cavity from immunized mice as well as Syk and Hck expression was increased, whereas in the in vitro assay, we observed that when the trophozoites of *N. fowleri* were opsonized with IgG anti-*N. fowleri* and interacted with PMN, the expression of Syk and Hck was also increased. We suggest that PMNs are activated via their FcγRIII, which leads to the elimination of the trophozoites in vitro, while in the nasal cavity, the adhesion and consequently infection are avoided.

## Introduction 

*Naegleria fowleri* is a protozoan causing primary amebic meningoencephalitis (PAM), an acute and fatal disease. Trophozoites adhere to the olfactory epithelial cells of the nasal cavity, cross the epithelium, and quickly migrate to the olfactory bulb and invade the brain (Marciano-Cabral & Cabral 2007; Rojas-Hernández et al. 2004a; Siddiqui et al. 2016). In recent years it has been possible to establish the protection model against *N. fowleri* infection, immunizing mice with total extract of *N. fowleri* co-administered with cholera toxin (CT) by intranasal routes; this immunization scheme induces 100% survival of mice that were challenged with 5 × 10^4^ lethal doses of live trophozoites of *N. fowleri* (Rojas-Hernández et al. 2004b).

Evidence shows that immunizations result in modulation of the response of production of IgA and IgG antibodies, as well as a response of inflammatory cells, particularly polymorphonuclear cells (PMNs) that are exuded into the lumen of the nasal cavity, where cells and antibodies are suggested to work together to prevent *N. fowleri* adhesion to the olfactory epithelium of the nasal cavity, which is a crucial step to avoid the infection (Carrasco-Yepez et al. 2014; Rojas-Hernández et al. 2004a). Recently, it was reported that in lumen of nasal cavity of mice infected with lethal doses of *N. fowleri* trophozoites, neutrophil extracellular traps (NETs) are released by PMNs near to the trophozoites; these NETs capture a certain amount of trophozoites; however, this mechanism is inefficient to prevent infection and death of mice (Carrasco-Yepez et al. 2019). In contrast, in vitro studies have shown that when *N. fowleri* were opsonized with IgG and they are interacted with PMNs, these amoebas were more susceptible to neutrophil activity (Contis-Montes De Oca et al. 2016), probably because the Fc receptors (FcR) on PMNs recognize the antigen–antibody complexes through the Fc region of the antibodies. It has been reported that this is a PMN activation mechanism that follows into effector functions of these cells (Bruhns 2012), and consequently, we suggest that such mechanism would eliminate the trophozoites, reducing their ability to infect. On the other hand, mice are only infected with trophozoites of *N. fowleri*; amoebas are found in the lumen of the nasal cavity covered with both IgA and IgG as well as being surrounded by many PMNs; however, all this events are not able to avoid the infection, probably because the PMNs are not being activated via the FcR as those PMNs from immunized mice (Carrasco-Yepez et al. 2014). The protective role of the FcγR has been studied in the protection against various pathogens like *Staphylococcus aureus*, where the immune complexes of IgG-*S. aureus* are recognized by the PMNs leading to the complement activation and phagocytosis (van Kessel et al. 2014), or with viruses like H1N1, where the interaction between anti-H1 monoclonal antibodies (mAbs) with the FcγR protects the mice from the H1N1 infection (DiLillo et al. 2014), or coronaviruses, where the binding of anti-SARS-CoV-2 mAbs with the FcγR contributes in the protection against SARS-Cov-2 in vivo (Schäfer et al. 2021).

The FcγRIII is present on most leukocytes and is involved in many of the effector functions of these cells (Bruhns & Jönsson 2015). Once the Fc receptor binds to the antigen–antibody complexes, a cross-linking of these receptors occurs on the membrane of the cell. The cytoplasmic region of the FcR has domains rich in tyrosine, which are phosphorylated by adapter molecules first by hemopoietic cell kinase (*Hck)* and later by spleen tyrosine kinases (*Syk*), which takes the phosphate groups and phosphorylates other proteins such as biochemical intermediates like phospholipase C γ (PLCγ) and phosphoinositide 3-kinase (PI3K), following with the activation of diacylglycerol (DAG) which activates enzymes like protein kinase C (PKC). The kinase enzyme phosphorylates nuclear factors like Elk-1, activator protein 1 (AP-1), and nuclear factor of activated T-cells (NFAT) that bind to different promoter regions of genes encoding for IL-2, IL-6, IL-8, integrins, and IFNγ (Sánchez-Mejorada & Rosales 1998). In this way, PMNs can carry out different effector functions such as phagocytosis, degranulation and activation of NADPH oxidase that led to respiratory burst (Löfgren et al. 1999), and induction of NETosis (Behnen et al. 2014).

When a pathogen makes contact with a epithelium, for example, the olfactory epithelium, these cells respond first as a physical barrier or by recognizing microbes with different receptors such as Toll-like receptors (TLRs), nucleotide-binding oligomerization domain (NOD)-like receptors, major histocompatibility complex (MHC) class I chain-related protein A (MICA), and MHC class I chain-related protein B (MICB), among others, resulting in the elimination of many pathogens (Vroling et al. 2008). Neutrophils also produce antimicrobial molecules as defensins that have been shown to possess the capability to recruit immunocompetent cells for the initiation of innate and adaptive immune responses (Lehrer et al. 2005). Recently, it has been suggested that FcγRIII is present in epithelial cells of the human nasal cavity where it activates these cells to produce cytokines in response to bacteria as an effector function of the epithelium (Golebski et al. 2019). It is important to notice that recent studies revealed the presence of the neonatal FcR (FcRn) co-localized with IgG in nasal epithelial cells of humans (Heidl et al. 2016), which mediates the IgG transcytosis, and it is responsible of the prolonged half-life of IgG (Kim et al. 2007).

Therefore, in this work, we analyzed the role of FcγRIII in the activation of PMNs, epithelial cells, and nasal passage cells from immunized and challenged mice with lethal dose of trophozoites of *N. fowleri* through recognition of antigen–antibody complexes, as well as the activation of adapter molecules such as *Hck* and *Syk*, which could be activating PMNs to participate in the elimination of the ameba.

## Material and method

### Animals

Male 6- to 8-week-old BALB/c mice were used in all experiments. All procedures were carried out under the standard: “NOM-062-ZOO-1999 Technical specifications for the production, care and use of laboratory animals SAGARPA,” the guide for the “Care and use of laboratory animals, National Research Council,” and it was approved by the ethical standards of the Institutional Animal Care and Use Committee (Number of Approval ESM.CICUAL-02/14–11-2018).

### Naegleria fowleri cultures and harvest

*Naegleria fowleri* ATCC30808 trophozoites were cultured axenically at 37 °C in Bacto Casitone medium (Difco, Le Pont-de-Claix, France) supplemented with 10% fetal bovine serum (GIBCO, Grand Island, NY) and 1% Antibiotic–Antimycotic Penicillin–Streptomycin 100 × (Corning, Cellgro, USA). Logarithmic phase trophozoites were harvested by centrifugation at 1500 g for 10 min, washed three times with phosphate buffer saline (PBS), and counted with a hemocytometer. The virulence of *N. fowleri* was reactivated according to the methodology reported by (Rojas-Hernández et al. 2004a). Only freshly harvested virulent amoebas were used for all experiments.

### Immunization and infection schedule

 Immunization and infection was carried out following the procedure previously reported by Rojas-Hernández et al. (2004b). BALB/c mice were immunized intranasally with 100 µg of *N. fowleri* lysates co-administered with 2 µg of cholera toxin (CT) as an adjuvant. Four doses were applied on days 1, 7, 14, and 21. Control mice received 30 µL of PBS. Mice were administered with 5 × 10^4^ live *N. fowleri* trophozoites in 30 µL of PBS on day 22. All mice were sacrificed on day 24.

### Subclasses of IgG anti-N. fowleri

The IgG and subclasses of IgG anti-*N. fowleri* levels in serum and nasal washes were evaluated using the ELISA technique following the procedures described (Carrasco-Yepez et al. 2014). The serum was added in a 1:100 dilution and the nasal washes in a 1:1 dilution in PBS-Tween 0.05% (PBS-T), and anti-IgG subclasses were added at rabbit anti-mouse IgG total (IgGt)/IgG1/IgG2a/IgG3 (1:500) (Thermo-Fisher). The results were analyzed using a Multiscan Ascent plate reader (Thermo Labsystems, Waltham, MA, USA) at 490 nm. Values were plotted using GraphPad 8.3.0 software.

### Specificity of anti-N. fowleri antibodies

The analysis of the anti-*N. fowleri* antibodies in nasal washes and serum were detected by Western blots according to the methodology reported by Rojas-Hernández et al. (2020). The proteins of the total extracts of *N. fowleri* (20 µg/well) were separated in 10% polyacrylamide gels by SDS-PAGE at 120 V for 90 min and transferred to a nitrocellulose membrane (400 mA, 60 min). The membranes were blocked with 2% of bovine serum albumin (BSA) (Research Organics) and incubated with serum (1:100) and nasal washes (1:1) and subsequently incubated with goat anti-mouse IgGt/IgG1/IgG2a/IgG3 (1:500) (Thermo Fisher). The protein recognition pattern was revealed with substrate solution (H_2_O_2_, 3.6 mM 4-chloro-1-napthol; Pierce).

### Fluorescence immunohistochemistry

To determine the presence of the IgG subclasses, as well as the presence of the FcγRIII in the nasal cavity of the BALB/c mice, 5-µm head slides of the different groups were obtained, following the procedures reported by (Carrasco-Yepez et al. 2019). Slides were blocked with 2% BSA for 1 h; samples were washed with PBS-T and subsequently incubated with rabbit anti-FcγRIII antibodies (1:500) (Abcam)/polyclonal hamster anti-*N. fowleri* (1: 250)/rabbit anti-mouse IgGt (1:1000)/rabbit anti-mouse IgG1 (1:500)/IgG2a (1:250)/IgG3 (1:500) (Thermo Fisher). Subsequently, the slides were incubated with secondary antibodies coupled to fluorochromes (Alexa 488® (green)/Alexa 647® (red)) (1:1000) and mounted with 4′,6-diamidino-2-phenylindole (DAPI) and VECTASHIELD (Vector Labs). Images were visualized using the Axioskop 2 mot plus confocal fluorescence microscope (Carl Zeiss, Mexico City).

### Anti-N. fowleri IgG purification

IgG anti-*N. fowleri* antibodies were purified from serum of immunized mice (with total amoeba extract) by affinity chromatography using an immobilized protein A-Sepharose CL-4B column (Sigma-Aldrich, St. Louis, MO, USA), following the methodology reported by Contis-Montes De Oca et al. (2016). The serum was diluted in equal parts with PBS and incubated on the column for 30 min. Protein-bound antibodies were eluted using 0.1 M glycine (pH 2.5) and collected for spectrophotometric analysis at 280 nm (SpectraPor; molecular weight cutoff, 10,000–14,000; Spectrum Medical Industries, Los Angeles, CA, USA.). The proteins were dialyzed in PBS, and the protein concentration was analyzed using the Bradford technique (Bradford 1976). The purified antibodies were used to opsonize live *N. fowleri* trophozoites in all in vitro experiments.

### Polymorphonuclear purification

PMNs were purified from the bone marrow of long bones of healthy mice and immunized and challenged mice. 1.5 mL of Roswell Park Memorial Institute (RPMI) 1640 1 × medium (Gibco, Invitrogen, USA) supplemented with 2% of fetal bovine serum (SFB) (Mexico, USDA approved) and 0.01% antibiotic-antifungal penicillin–streptomycin 100 × (Corning, Cellgro, USA) were passed through the bone using an insulin syringe, the bone marrow was recovered and disintegrated to later be placed on Histopaque gradients (Histopaque 1077/Histopaque 1119), and the methodology reported by (Wardini et al. 2010) was followed. Cell viability was determined by trypan blue exclusion method, and purity was checked by Turk staining in a Neubauer chamber.

### Opsonization of N. fowleri

Live *N. fowleri* trophozoites were opsonized with 10 µg of purified mice IgG antibodies for 3 min. Subsequently, centrifuged 1500 g/5 min to remove unbound antibodies. The opsonized amoebas were interacted with the purified PMNs from immunized mice.

### Hamster IgG anti-N. fowleri purification

Hamsters were immunized intraperitoneally with 100 µg of *N. fowleri* lysates. Four doses were applied on days 1, 7, 14, and 21. Later, animals were sacrificed, and serum was obtained. IgG anti-*N. fowleri* was purified as described above.

### Interaction of PMNs with opsonized or non-opsonized amoebas

PMNs cells were adjusted to 1 × 10^6^ (0.2 mL) and were incubated on glass coverslips treated with 0_001% poly-L-lysine (Sigma-Aldrich) in a 24-well plate. Cells were placed at the bottom of the wells for 30 min at 37 °C, 5% CO_2_ to allow cell adhesion. Subsequently, unopsonized or opsonized (10 µg of human IgG) *N. fowleri* trophozoites (1 × 10^6^) in RPMI were added (PMNs/trophozoites ratio of 10:1) for 15, 30, and 60 min. Some wells of the same culture plate were used to incubate PMNs with a polyclonal rabbit anti-mice FcγRIII antibody (Invitrogen) in order to block the FcγRIII receptor of PMNs before interaction with trophozoites. The samples were fixed with 2% paraformaldehyde (PFA) at 37 °C/10 min. The cells were permeabilized with 0.05% Triton X-100 (Research Organics, Cleveland, OH, USA) for 10 min and blocked with 2% BSA (Research Organics) to proceed with the staining. Antibodies (rabbit anti-FcγRIII) (1:500) (Abcam, USA), goat anti-IgG (1:500) (Abcam), and anti-*N. fowleri* (polyclonal IgG hamster) (1: 100) were used for 24 h at 4 °C. Subsequently, the cells were incubated with fluorochrome-coupled antibodies (Alexa 488 ® (green)/Alexa 647 ® (red)/anti-mouse IgG Texas Red (orange) (1: 1000)). DNA was stained using DAPI (blue) with VECTASHIELD (Vector Labs). The cells were visualized using the Axioskop 2 mot plus confocal fluorescence microscope (Carl Zeiss, Mexico City).

### Cell viability

To analyze the viability of PMNs and *N. fowleri*, trypan blue staining was performed. Live opsonized or non-opsonized *N. fowleri* trophozoites were interacted for 15, 30, and 60 min with blocked or unblocked FcγRIII purified PMNs, as described above. After the interaction, 0.4% trypan blue was added for 1 min. The cells were counted in 10 visual fields under a microscope (× 400, magnification). Values were plotted using GraphPad 8.3.0 software (GraphPad Software, Inc., San Diego, CA).

### Purification of epithelial cells and nasal passages from the nasal cavity

Epithelial cells and nasal passages were purified from healthy mice and immunized and challenged mice. The heads of mice were obtained, from which the skin was removed, and a cut was made to remove the nasal cavity at eye level. Tissues were disrupted with 3 mL of RPMI-1X medium (RPMI 1640 (sigma Aldrich) + 1.5 mM EDTA (Sigma Aldrich) + 1 mM DTT (Sigma Aldrich) + 5% SFB (Gifco)). Isolation was performed by gradient Percoll density centrifugation method (70%-40%-20% Percoll) (Sigma Aldrich). Finally, centrifuged at 450 g/30 min. The cells were recovered from the interface, and washes were carried out with PBS to eliminate the Percoll residues. The purified cells were used for RNA extraction, as well as detection of signaling factors by Western blot.

### Total RNA extraction

The extraction was carried out from cells of lamina propria, and nasal cavity of BALB/c mice and mRNA was isolated with the TRIzol® Reagent technique, following the procedure reported by Bandala et al. (2021) with modifications. The isolation of the total RNA was carried out using a mixture of phenols (TRIzol), isopropanol, and ethanol at 80%, dried in a SpeedVac, and resuspended in RNase-free water. Its integrity was verified by electrophoresis in a 1.5% agarose gel labeled with an ethidium bromide analog (Midori Green), and its purity was determined by the difference of readings 260–280 nm of the spectrophotometer. The total RNA concentration was 500 ng. Before proceeding with the reverse transcription, it was stored at − 80 °C.

### cDNA synthesis

The synthesis kit “SensiFAST cDNA Synthesis Kit/Bioline” was used according to the manufacturer’s instructions with 4 µL of total RNA for each sample. Reverse transcription was carried out by incubating at the following temperatures: 10 min/25 °C, 15 min/42 °C, and 5 min/85 °C final for enzyme inactivation.

### Real-time polymerase chain reaction

The relative expression of the mRNA of the genes was determined using probes from the mouse transcriptome library (Mouse Universal Probe Library), and a Techne Prime Pro-48 Real-Time qPCR thermocycler, with a TaqMan-type reaction mixture of the brand “SensiFAST Probe No-ROX kit/Bioline.” The oligo sequences of the primers (sense and antisense) were designed with Probe Finder software version 2.45 (http://www.universalprobelibrary.com). *Syk* Gen (Forward 5′-GTGCAATACTTTCCCTTCGTG-3′, Reverse 3′-AAGACCAATGGAAAATTCCTGAT-5′), *Hck* Gen (Forward 5′-CTGCAGCGACTTCATCAGG-3′, Reverse 3′-GGGCAGTTTGGAGAAGTGTG-5′), *FcγRIII* Gen (Forward 5′-CAGAAACACCCGCTGAGG-3′, Reverse 3′-CAGCGACCCTGTAGATCTGG-5′), 18 s (Forward 5′-CTCAACACGGGAAACTCAC-3′ Reverse 3′-CGCTCCACCAACTAAGAACG-5′).

The reaction mixture was prepared according to the manufacturer’s protocol for the thermal cycler (Techne Prime Pro-48) with the following protocol: 2:01 min/95 °C, followed by 10 s/95 °C, 20 s/60 °C, and 10 s/72 °C; these last 3 temperatures were repeated for 45 cycles. Before the experiments, a standardization curve of 6 serial dilutions was included for each gene to establish a homogeneous analysis for all genes. Each experiment was analyzed in triplicate under the same conditions with Pro Study Software Techne (Bibby Scientific-US).

*Syk*, *Hck*, *and FcγRIII Western blot analysis.*

The detection of cell signaling factors of *Hck* and *Syk*, as well as FcγRIII in epithelial cells, nasal passages, and PMNs of control and immunized mice, was carried out employing Western Blot, following the methodology described above. Rabbit anti-mouse *Hck* (1: 250)/rabbit anti-mouse *Syk* (1:250)/polyclonal anti-mouse FcγRIII (1:100) antibodies were used. The protein recognition pattern was revealed with solution (H_2_O_2_, 3.6 mM 4-chloro-1-napthol; Pierce).

### Statistical analysis

Statistical analyses were performed using Prism GraphPad 8.3.0 software (GraphPad Software, San Diego, CA, USA) Two-way analysis of variance unpaired test with Tukey post-test. *P* values of *P* < 0.05 were considered significant.

## Results

### Immunization with total extract of N. fowleri plus CT increases levels of specific IgG subclasses

**Fig. 1:**
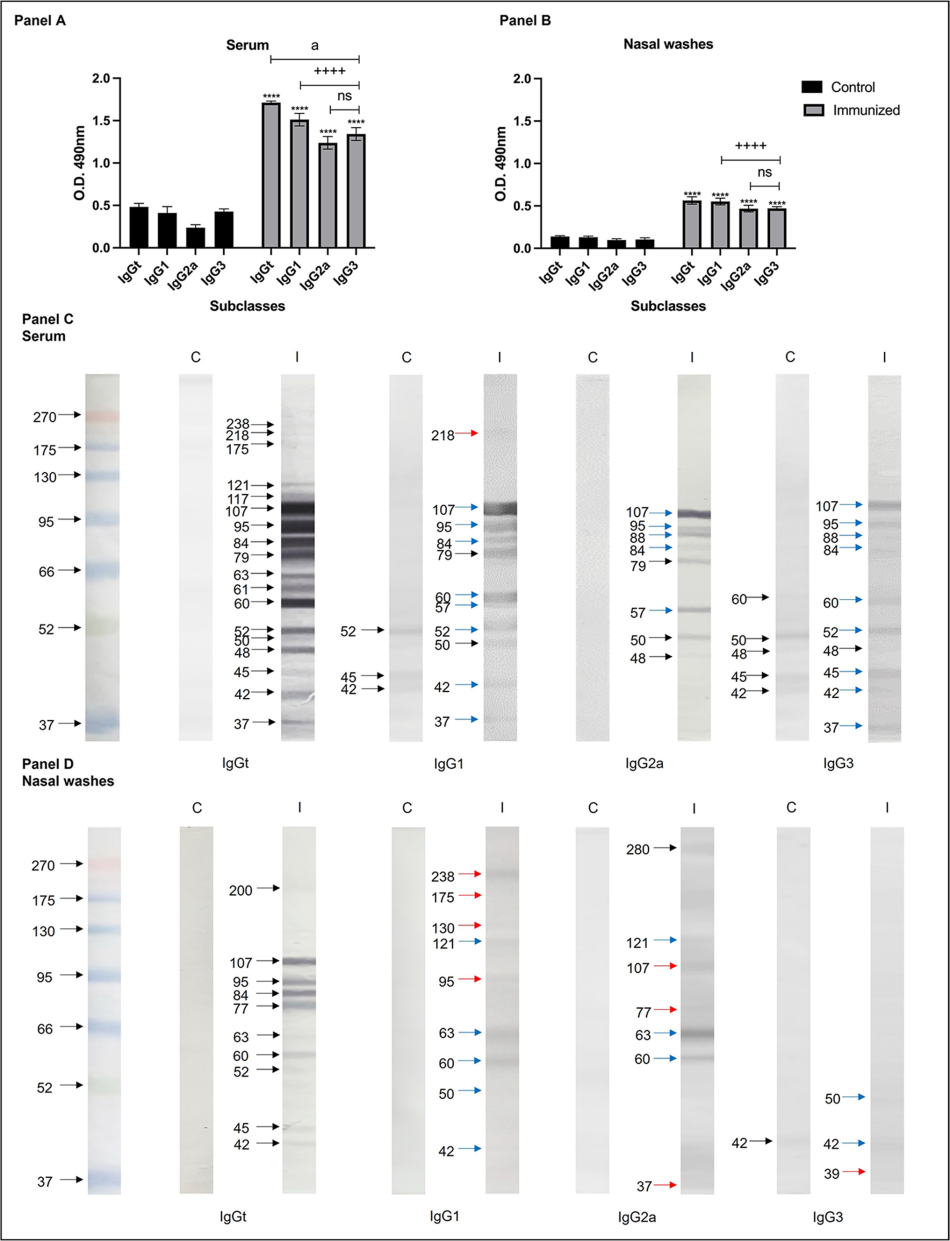
Response of anti-*N. fowleri* IgG subclasses in immunized mice. BALB/c mice were immunized via intranasal with 100 µg of total *N. fowleri* extract co-administered with 2 µg CT for four times at 7-day intervals and subsequently challenged with live *N. fowleri* trophozoites. Subclasses of IgG anti-*N. fowleri* were analyzed in **A** serums and **B** nasal washes of the groups by means of ELISA. Total extracts of *N. fowleri* were subjected to electrophoresis in SDS-PAGE 10% polyacrylamide gels (120 V, 90 min) and transferred to a nitrocellulose membrane (400 mA, 60 min). Healthy mice and immunized mice with total extract of *N. fowleri* plus CT and challenged with live trophozoites of *N. fowleri* were incubated with **C** serums and **D** nasal washes. Protein recognition pattern was revealed using H_2_O_2_, 3.6 mM 4-chloro-1-napthol. Individual samples were analyzed in triplicate. The data expressed absorbance values at 490 nm mean ± SD of each experimental group (*n* = 5). Four asterisks indicate significant difference of *P* < 0.0001 between control and immunized, four plus signs indicate significant difference of *P* < 0.0001 between subclasses in immunized and challenged mice, and *a* indicates significant difference of *P* < 0.0001 between serum and nasal washes. C control group, I immunized and challenged group

The levels of IgG1, IgG2a, and IgG3 present in serum and nasal washes of immunized and control mice were determined by ELISA (Fig. 1A–B). We observed that in both serum and nasal washes samples, the three analyzed subclasses and IgGt of immunized mice were significantly higher than control mice (*P* < 0.0001) (Fig. 1A–B), while IgGt was significantly higher than the three analyzed subclasses (*P* < 0.0001). IgG1 subclass was significantly higher than IgG2a and IgG3 in the immunized mice (*P* < 0.0001). However, nasal washes values were significantly lower than serum samples.

We determined the pattern of recognition of *N. fowleri* antigens by the different IgG isotypes analyzed, as well as IgGt from the samples of the immunized and control mice using the immunoblot technique (Fig. 1C–D). Firstly, IgGt from sera of immunized mice recognized different bands of *N. fowleri* of broad molecular weight (238 to 37 kDa). Particularly, the bands from 121 to 37 kDa were recognized with a great intensity. IgG1 recognized bands with molecular weight from 218 to 37 kDa. The bands of 218, 107, 95, 84, 79, 60, 52, 50, 42, and 37were recognized by IgG1 with a lower intensity than the recognition given by IgGt. IgG1 and IgG2a recognized less quantity of bands with a molecular range from 107 to 48 kDa. The bands with molecular weight of 107, 95, 84, 79, 57, and 50 were recognized by both isotypes, while the 88- and 48-kDa bands were recognized only by IgG2a. IgG3 was able to recognize bands from 107 to 37 kDa. Particularly, the bands recognized by the three subclasses were those of 107, 95, 84 kDa. The 60-, 52-, 42-, and 37-kDa bands were recognized by IgG1 and IgG3, while the 88- and 48-kDa bands were recognized by IgG2a and IgG3. IgG1 was the only subclass that recognized the 218-kDa band (Fig. 1C) (red arrow).

When we analyzed the nasal washes samples, we observed that IgGt could recognize bands with molecular weights between 200 and 42 kDa, increasing the recognition towards bands of 107, 95, 84, 77, and 60 kDa. IgG1 recognized bands from 238 to 42 kDa. The 95-, 63-, 60-, and 42-kDa bands were found in both IgGt and IgG1. However, the 238-, 175-, 130-, and 95-kDa bands were only recognized by the IgG1 subclass (Fig. 1D) (red arrows), while IgG2a recognized seven bands between 280 and 37 kDa. The 121-, 63-, and 60-kDa bands were recognized by both IgG1 and IgG2a. Compared to the other subclasses, IgG2a recognized the 107- and 77-kDa bands (Fig. 1D) (red arrow). IgG3 was the subclass where less number of bands were recognized, those of 50, 42, and 39 kDa. The 42-kDa band was recognized by both IgG1 and IgG3, while the 39-kDa band was recognized only by IgG3 (red arrow). It should be noted that after the immunizations, a significant increase was obtained regarding the number of detected bands as well as their intensity compared to the recognize given by the antibodies from control groups (Fig. 1C–D) (blue arrows). In both types of samples, serum and nasal washes, IgG1 was the subclass that had the highest recognition regarding the number of bands.

### Detection of IgG subclasses in nasal epithelium of immunized mice

**Fig. 2:**
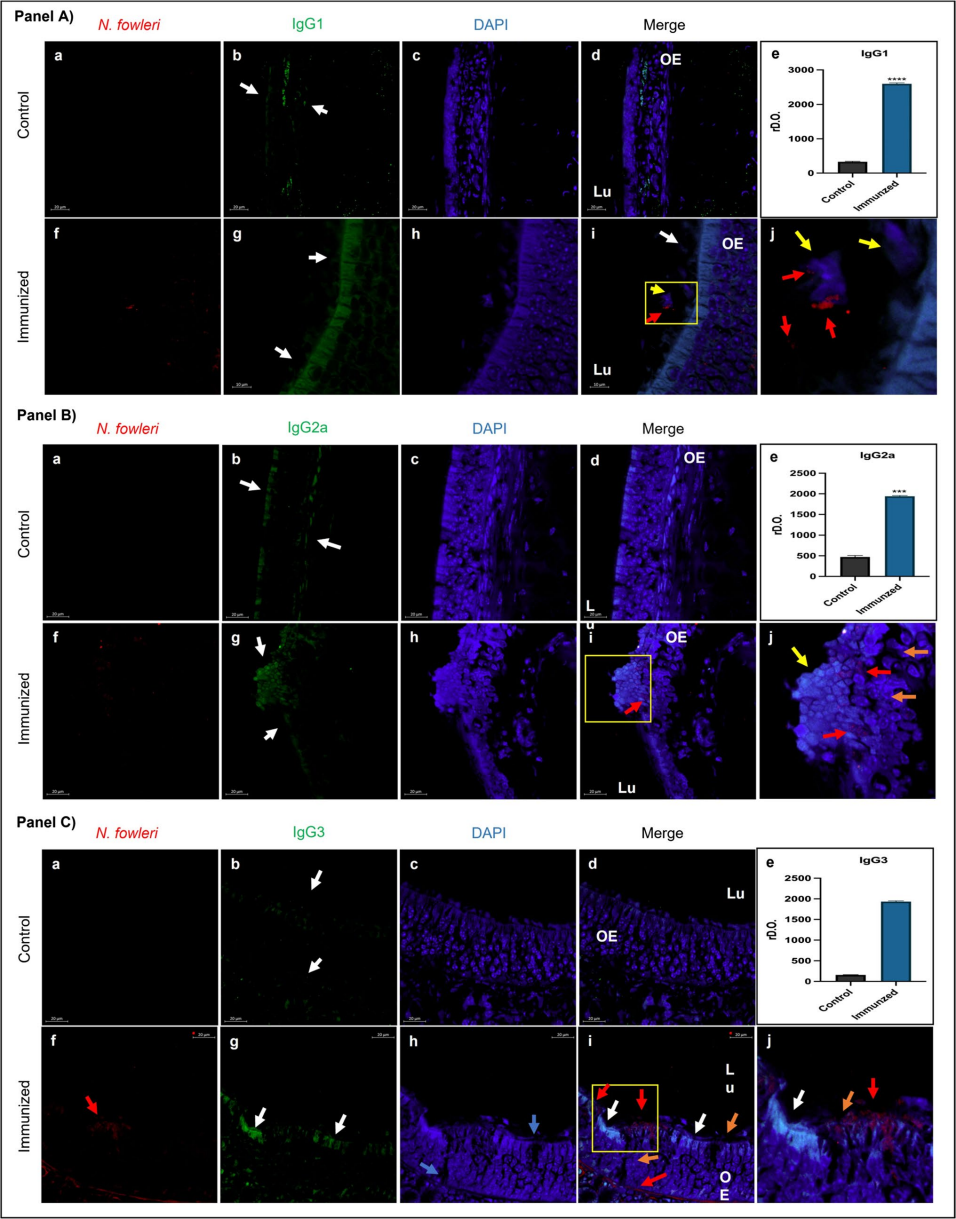
Increase in the presence of IgG subclasses in immunized mice. BALB/c mice were immunized with total *N. fowleri* extract plus CT and subsequently challenged with live *N. fowleri* trophozoites. Histological slides of the nasal cavity (5 µm) were made and stained with anti-*N. fowleri* (red); anti-IgG1, anti-IgG2a, and anti-IgG3 (orange); and DNA with the DAPI (blue). After the immunization scheme, it is observed that the presence of the subclasses is increased in the nasal mucosa, preventing the adhesion of *N. fowleri* to the epithelium. The analysis of the optical density of the positive staining for the IgG subclasses was performed using the ImageJ software, where the significant increase (*P* < 0.05) was obtained in the mice that were immunized and challenged compared to the control group. Images and graphs show the mean ± SD of three independent experiments (*n* = 3). Three asterisks indicate significant difference of *P* < 0.001, and four asterisks indicate significant difference of *P* < 0.0001. Scale bars 20 µm. Lu lumen of the nasal cavity, OE olfactory epithelium

The presence and distribution of IgG subclasses in the nasal cavity were analyzed by immunohistochemistry technique (Fig. 2). Slides of 5 µm were obtained and incubated with specific anti-*N. fowleri* (red) and anti-IgG1/anti-IgG2a/anti-IgG3 (green) antibodies, and the DNA was stained with DAPI (blue). Samples were observed under a confocal microscope.

For control mice, IgG1 stain was slight in both apical and basal regions of the olfactory epithelium (Fig. 2A, b) (white arrows). In contrast, in immunized mice, the stain for IgG1 increased significantly (*P* < 0.0001), and it was observed from the basolateral to the apical region of the olfactory epithelium (Fig. 2A, g) (white arrows). In the merge, we observed in the lumen the presence of *N. fowleri* (red arrows) surrounded by inflammatory cells (yellow arrow), and apparently, the trophozoites were destroyed (Fig. 2A, i, j) (red arrows).

In contrast with IgG1, the stain for IgG2a in control mice was observed in the basal and apical region of the olfactory epithelium (Fig. 2B, b) (white arrows). The increment of IgG2a was more intense, and it was distributed mainly in the olfactory epithelium and inflammatory exudate of immunized mice (Fig. 2B, g) (white arrows). In the merge, we can clearly observe detritus of *N. fowleri* immersed in this inflammatory exudate (Fig. 2B, i, j) (red arrows), and also we can observe along with the exudate a large amount of IgG2a together with the inflammatory cells. In Fig. 2B, i, j, the orange arrows indicate that the epithelium appears to be ruptured to allow the inflammatory exudate to escape, and IgG2a probably escapes into the lumen through this rupture. Densitometric analysis was performed, where the IgG2a stain in the immunized mice was significantly higher than control mice (*P* < 0.001).

Like the other two analyzed IgG subclasses, the stain of IgG3 in control mice was diffuse and poorly distributed in the basal and apical regions in the olfactory epithelium (Fig. 2C, b) (white arrows), while in the immunized group, the stain is found distributed from the basolateral towards the apical region of the olfactory epithelium, with greater presence in the apical region (Fig. 2C, g) (white arrows). In the merge, we can also observe detritus of *N. fowleri* in the apical region of the olfactory epithelium; however, the stain is diffused and surrounded by IgG3 positive cells (Fig. 2C, i j) (red arrows). Interestingly, it can be observed the shedding of large numbers of epithelial cells along with large staining for IgG3. The pathways through which the inflammatory exudate could escape are observed (Fig. 2C, i, j) (orange arrows). The densitometric analysis confirms the significant increment in the stain of the IgG3 due to the immunization scheme compared with the control group (*P* < 0.0001).

### Presence and modulation of the FcγRIII expression in the nasal cavity

**Fig. 3:**
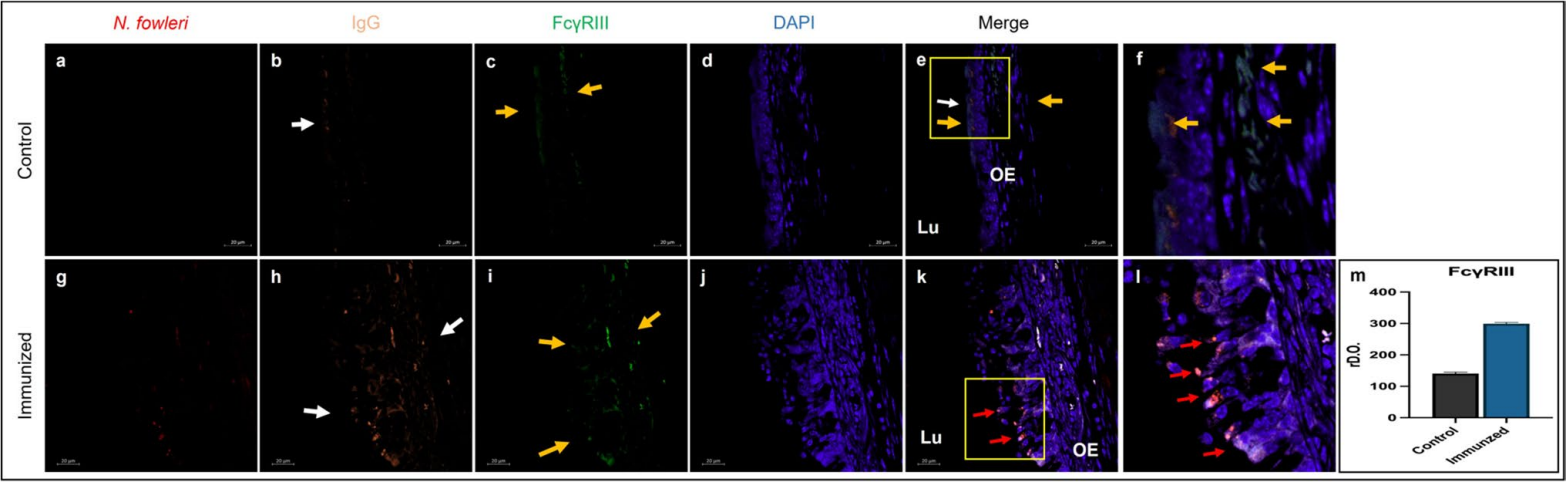
Increment in the presence of FcγRIII and IgG in immunized mice. BALB/c mice were immunized with total *N. fowleri* extract plus CT and subsequently challenged with live *N. fowleri* trophozoites. Histological slides of the nasal cavity (5 µm) were made and stained with anti-*N. fowleri* (red), anti-IgG (orange), anti-FcγRIII (green), and the DNA with DAPI (blue). After immunization, IgG antibodies show an increment in immunized and challenged mice compared to the control group (h), the same behavior is observed with the FcγRIII (i). Strong labeling for the receptor and the antibody is seen in the apical region of the epithelium, where *N. fowleri* attempts to adhere to epithelial cells. Optical density analysis of the positive staining for FcγRIII was performed using ImageJ software, where the significant increase (*P* < 0.05) was obtained in the mice that were immunized and challenged compared to the control group. Images and graphs show the mean ± SD of three independent experiments (*n* = 3). Three asterisks indicate significant difference of *P* < 0.001. Scale bars 20 µm. Lu lumen of the nasal cavity, OE olfactory epithelium

Control and immunized mice were used for detecting the presence of FcγRIII in the nasal cavity of BALB/c mice. Histological sections of 5 μm of mouse heads were obtained, which were incubated with anti-*N. fowleri* (red), anti-IgG (orange), anti-FcγRIII (green) antibodies and observed in a confocal microscope. The obtained images are shown in Fig. 3, where in the control group it is observed a slight stain for IgG which is distributed in the apical region of the olfactory epithelium (Fig. 3, b) (white arrows), while FcγRIII was detected both in the basal and apical regions of the olfactory epithelium (Fig. 3, c–e–f) (yellow arrows). The merge shows the co-location of the FcγRIII and IgG in the apical region of the olfactory epithelium (Fig. 3, e) (white arrow). It is important to note that the stain of FcγRIII in immunized mice is mostly distributed in the basal side towards the apical region of the olfactory epithelium finding an increase regarding the stain intensity compared to the control group (Fig. 3, i) (yellow arrows). IgG stain also appears to be incremented for the same regions (Fig. 3, h) (white arrows). In the merge, the stains for IgG and FcγRIII are co-located in the apical region of the olfactory epithelium; this suggests that there is immunologic activity against the amoeba (Fig. 3, k–l) (red arrows). The stain for *N. fowleri* which is observed inside the epithelium was diffuse, suggesting that amoebas may have been destroyed by the presence of inflammatory cells (Fig. 3, k) (white arrow). Optical density analysis was performed for the FcγRIII where a significant increase of these receptor was observed in immunized mice with respect to the control group (*P* < 0.001) (Fig. 3, m).

### *Recognition of immune complexes by FcγRIII of PMNs *in vitro

**Fig. 4:**
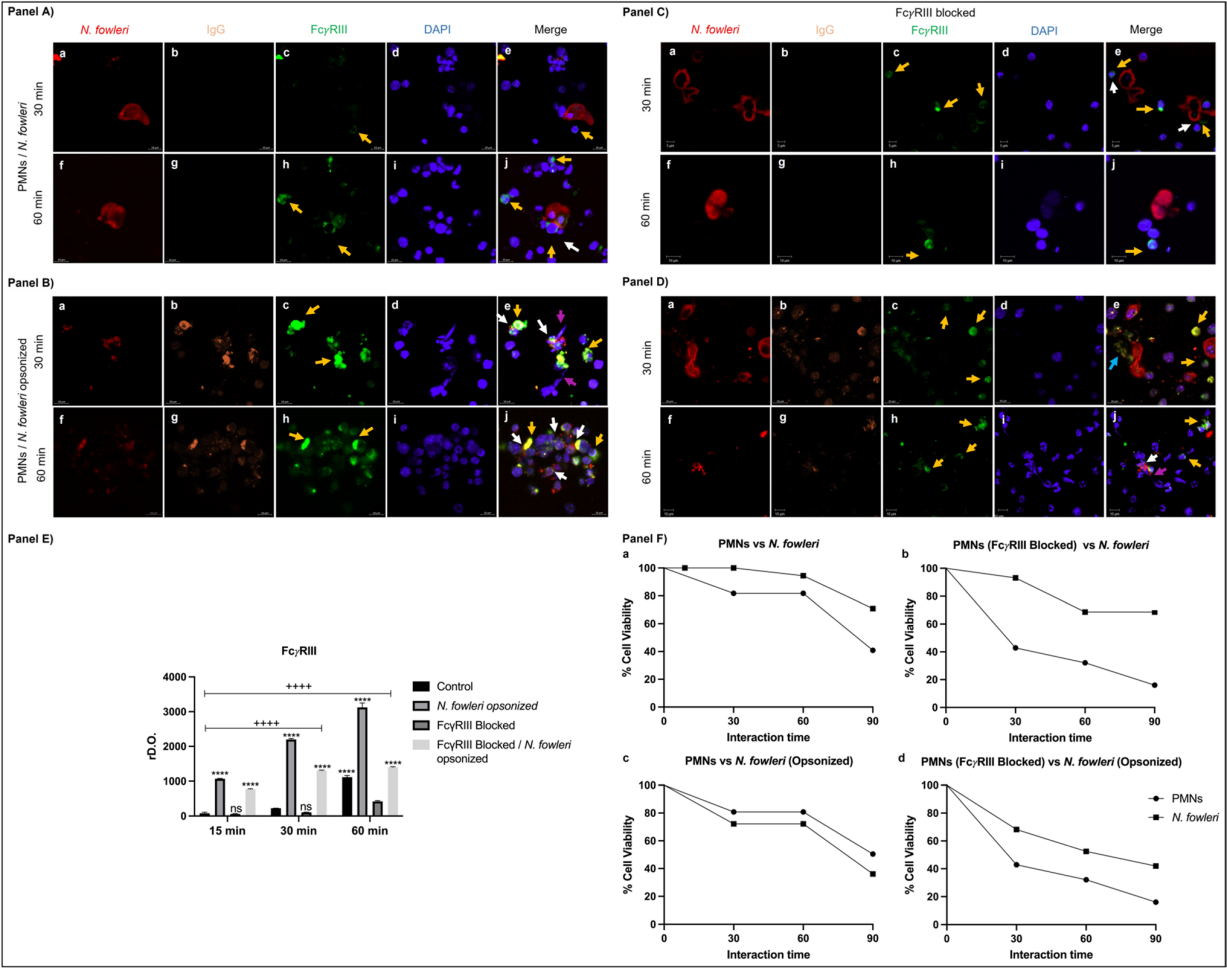
Opsonization of *N. fowleri* induces its destruction by PMNs. PMNs from immunized and challenged mice were interacted with live opsonized *N. fowleri* trophozoites with IgG or without opsonization at different times (15, 30, and 60 min). A group of PMNs was incubated with anti-FcγRIII to block FcγRIII. IgG opsonized amoebas were surrounded by several PMNs at 30 min, where the release of extracellular traps was also observed. After 60 min, no integrity was observed in *N. fowleri*. Densitometric analysis reveals a significant increment in FcγRIII expression when amoebas are opsonized with IgG (*P* < 0.05). In addition, the cell viability was counted in the interactions where it is observed that the PMNs that recognize the opsonized amoebas can eliminate most of them after 60 min of interaction. Images and graphs show the mean ± SD of three independent experiments (*n* = 3). Four asterisks indicate a significant difference of *P* < 0.0001 between unopsonized and opsonized amoebas, and four plus signs indicate a significant difference of *P* < 0.0001 between interaction times. Scale bars 10 µm

Firstly, the PMNs obtained from bone marrow of immunized and challenged mice had approximately an 85% of purity with a viability of 99%. Thus, to demonstrate the role of the FcγRIII receptor in the recognition of immune complexes and consequently possible destruction of *N. fowleri*, interactions were performed between these PMNs with opsonized and non-opsonized *N. fowleri* trophozoites (Fig. 4). We observed that at 30 min, PMNs interacted with non-opsonized trophozoites had a slight FcγRIII stain in the cellular membrane (Fig. 4A, c, e) (yellow arrow). In the merge, we observed a few PMNs near to *N. fowleri* trophozoites (Fig. 4A, e) (yellow arrows). After 60 min, we detected an increase in the FcγRIII stain in the cellular membrane of the PMNs (Fig. 4A, h, j) (yellow arrows) as well as a greater number of PMNs in contact with the amoeba (Fig. 4A, j) (white arrow). However, it is not observed that the amoebas present damage, suggesting that the PMNs are not being effective in the elimination of the amoeba.

Regarding the interaction between PMNs and opsonized trophozoite*s,* we observed that at 30 min, the stain for FcγRIII has been significantly increased in the PMNs cellular membrane (Fig. 4B, c). In the merge, a greater number of PMNs are observed surrounding the opsonized amoeba (Fig. 4B, e) (white arrows); furthermore, extracellular traps-like are found in the contact areas between the trophozoites and the PMNs (Fig. 4B, e) (purple arrows). It is important to highlight that in some areas where the stain of FcγRIII was intense, extracellular traps-like were detected (Fig. 4B, e) (yellow arrows). After 60 min, the stain for FcγRIII is highly intense (Fig. 4B, h) (yellow arrows), whereas the stain for *N. fowleri* was diffuse, suggesting that the amoebas are being damaged (Fig. 4B, f). In the merge, we observed a great number of PMNs in association with what appears to be amoeba debris (Fig. 4B, j), suggesting that amoebas are being destroyed by the activity of PMNs after recognition of immune complexes by FcγRIII.

To verify whether the destruction of trophozoites was mediated by FcγRIII, we performed interactions of PMNs previously incubated with an anti-FcγRIII antibody to block the FcγRIII receptor with opsonized and non-opsonized *N. fowleri* trophozoites. These interactions were carried out at the same times considered in the previous experiment.

When PMNs were interacted with non-opsonized amoebas, we could observe a low number of PMNs at 30 min with a slight stain for FcγRIII (Fig. 4C, c, e) (yellow arrows). In the merge, we observed PMNs in proximity to *N. fowleri* without making contact with the amoeba (Fig. 4C, e) (white arrow). After 60 min, the stain for FcγRIII was observed more intense than the interactions of 30 min (Fig. 4C, h, j); however, not apparent activity of PMNs against amoebas was observed.

When we opsonized the trophozoites and interacted with PMNs pre-incubated with anti-FcγRIII at 30 min, it was observed that the stain for FcγRIII in the PMNs surface was increased, both at 30 and 60 min (Fig. 4D, c, h) (yellow arrows). In the merge, we did not observe damage in *N. fowleri*; however, it seems that the PMNs were damaged since the FcγRIII stain was diffuse (Fig. 4D, e) (blue arrow). At 60 min of interaction, some PMNs showed FcγRIII on their surface (yellow arrows) (Fig. 4D, h, j); however, the stain for both *N. fowleri* and PMNs was observed heterogeneous suggesting lost of cell integrity. In addition, few PMNs were contacting the amoeba (Fig. 4D, j) (white arrow), PMNs that seem to be releasing extracellular tramps in that area (Fig. 4D, j) (purple arrow).

The densitometric analysis of the FcγRIII stain was performed after the interactions, where the graphed results are shown in Fig. 4E. It is observed that the expression of FcγRIII is dependent on the interaction time between PMNs and *N. fowleri* as well as whether the amoebas are opsonized or not, since when PMNs interact with amoeba opsonized, the stain for FcγRIII increase compared with the rest of the groups (*P* < 0.05), being at 60 min when the receptor is most expressed. We also can observe that the block effect given by the anti-FcγRIII influenced the decreased expression of such receptor, mainly in those PMNs that were interacted with non-opsonized trophozoites.

The viability of trophozoites and PMNs during interactions was analyzed (Fig. 4F). After 1 h of interaction between PMNs and unopsonized *N. fowleri* trophozoites, a 40% of the PMNs survived, while *N. fowleri* trophozoites had 80% survival (Fig. 4F, a). When the amoeba was opsonized and interacted with PMNs, a change was observed, since 50% of the PMNs survived and a 40% of survival of opsonized *N. fowleri* was obtained (Fig. 4F, b), suggesting that the PMNs are not capable to eliminate trophozoites by themselves; they need to be opsonized for the immune complexes to be recognized by FcγRIII.

Regarding the interactions carried out with the PMNs that were previously incubated with anti-FcγRIII and amoebas without opsonization, we found that 80% of the PMNs died, and 70% of *N. fowleri* trophozoites survived (Fig. 4F, c). When the trophozoites were opsonized and had interacted with pre-incubated PMNs, survival of 20% of PMNs and 40% of *N. fowleri* was obtained (Fig. 4F, d).

### Expression of FcγRIII, Syk, and Hck genes and proteins in the nasal mucosa

**Fig. 5:**
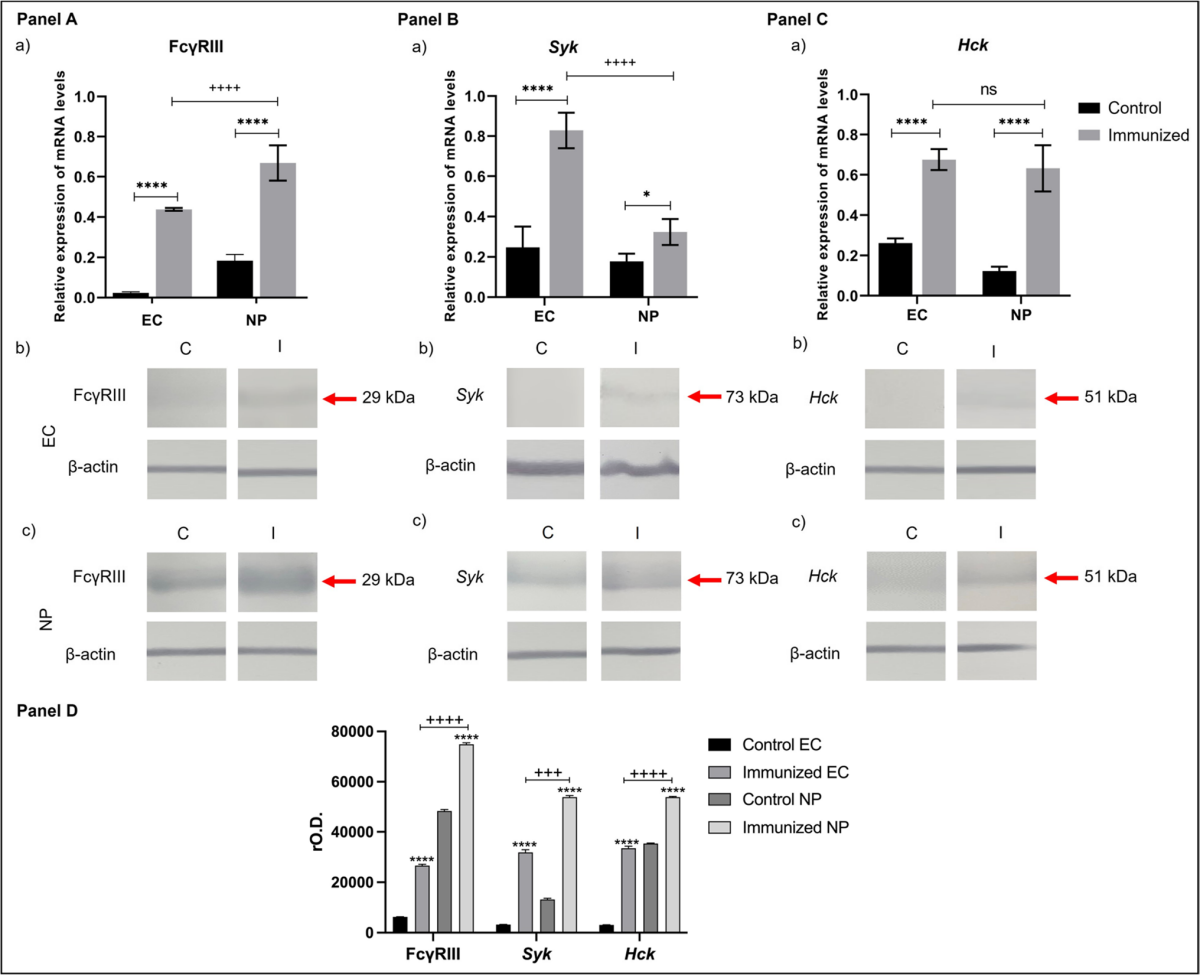
Expression of FcγRIII, *Syk*, and *Hck* in epithelial cells and nasal passages. Epithelial cells and nasal passages from healthy mice and immunized and challenged mice were purified, and the genes of **A** FcγRIII, **B** *Syk*, and **C** *Hck* were analyzed by RT-PCR and Western blot. The results show that after immunizations, mRNA expression levels increased for the three genes analyzed. Protein expression is also increased after immunizations, both in epithelial cells and nasal passages of mice immunized with total extract of *N. fowleri* and challenged with live trophozoites. The protein expression of FcγRIII, *Syk*, and *Hck* was higher in nasal passages compared to epithelial cells. The graphs show the mean ± SD of three independent experiments (*n* = 3). Four asterisks indicate significant difference of *P* < 0.0001 between control mice and immunized and challenged mice, and four plus signs indicate significant difference of *P* < 0.0001 between epithelial cells and nasal passages

To verify the presence of FcγRIII, as well as its possible role in the protection against infection by *N. fowleri*, we evaluated the expression of the FcγRIII, as well as *Syk* and *Hck* kinases in epithelial cells of the mucosa and nasal passages of control and immunized mice by RT-PCR and Western blot. The results are shown in Fig. 5. We observed that the three analyzed genes were expressed in epithelial cells and nasal passages, both in control and immunized mice, but the expression of these genes was higher in immunized mice than control group (*P* < 0.0001) (Fig. 5A, B, C). The FcγRIII gene is significantly more expressed in nasal passages than in epithelial cells (*P* < 0.0001) (Fig. 5A, a). The *Syk* gene is significantly higher in epithelial cells compared to nasal passages (*P* < 0.0001) (Fig. 5B, a). However, there is no significant difference of *Hck* gene in both epithelial cells and nasal passages (*P* < 0.05) (Fig. 5C, a).

When we performed a Western blot analysis, it confirmed the presence of the proteins FcγRIII, *Syk*, and *Hck* in epithelial cells and nasal passages of control and immunized mice. FcγRIII with a rMW 29-kDa band was detected with higher intensity in epithelial cells of immunized mice than control mice (*P* < 0.0001) (Fig. 5A, b). Meanwhile, in nasal passages, this band was detected with a significantly higher intensity in immunized mice compared with the control group as well as compared with the epithelial cells from immunized mice (*P* < 0.0001) (Fig. 5A, c).

We did not found expression of protein bands in epithelial cells of control mice for *Syk*. However, a band of 73 kDa appears in mice that were immunized (Fig. 5B, b), whose molecular weight corresponds to that reported for *Syk*. In nasal passages, the 73 kDa band is observed in control mice, and the band is more intense in immunized mice (Fig. 5B, c).

Regarding *Hck*, the Western blot did not show bands for *Hck* in epithelial cell of control group; in contrast, after immunizations and challenge, a faint band of 51 kDa is found (Fig. 5C, b). In nasal passages, a band of 51 kDa is lightly observed, which corresponds to the molecular weight reported for *Hck*, while in nasal passages of immunized and challenged mice, the band of 51 kDa was more intense (Fig. 5C, c).

Finally, the densitometric analysis of the bands obtained in the Western blot was carried out, where we can corroborate that after immunizations, the expression of the proteins increases significantly for FcγRIII and for its signaling molecules, *Syk* and *Hck*, compared to control mice (*P* < 0.0001). In all cases, the nasal passages had the higher protein expression compared to the epithelial cells from immunized mice group (*P* < 0.0001).

### Expression of FcγRIII, Syk, and Hck genes and proteins in PMNs interacted with N. fowleri

**Fig. 6:**
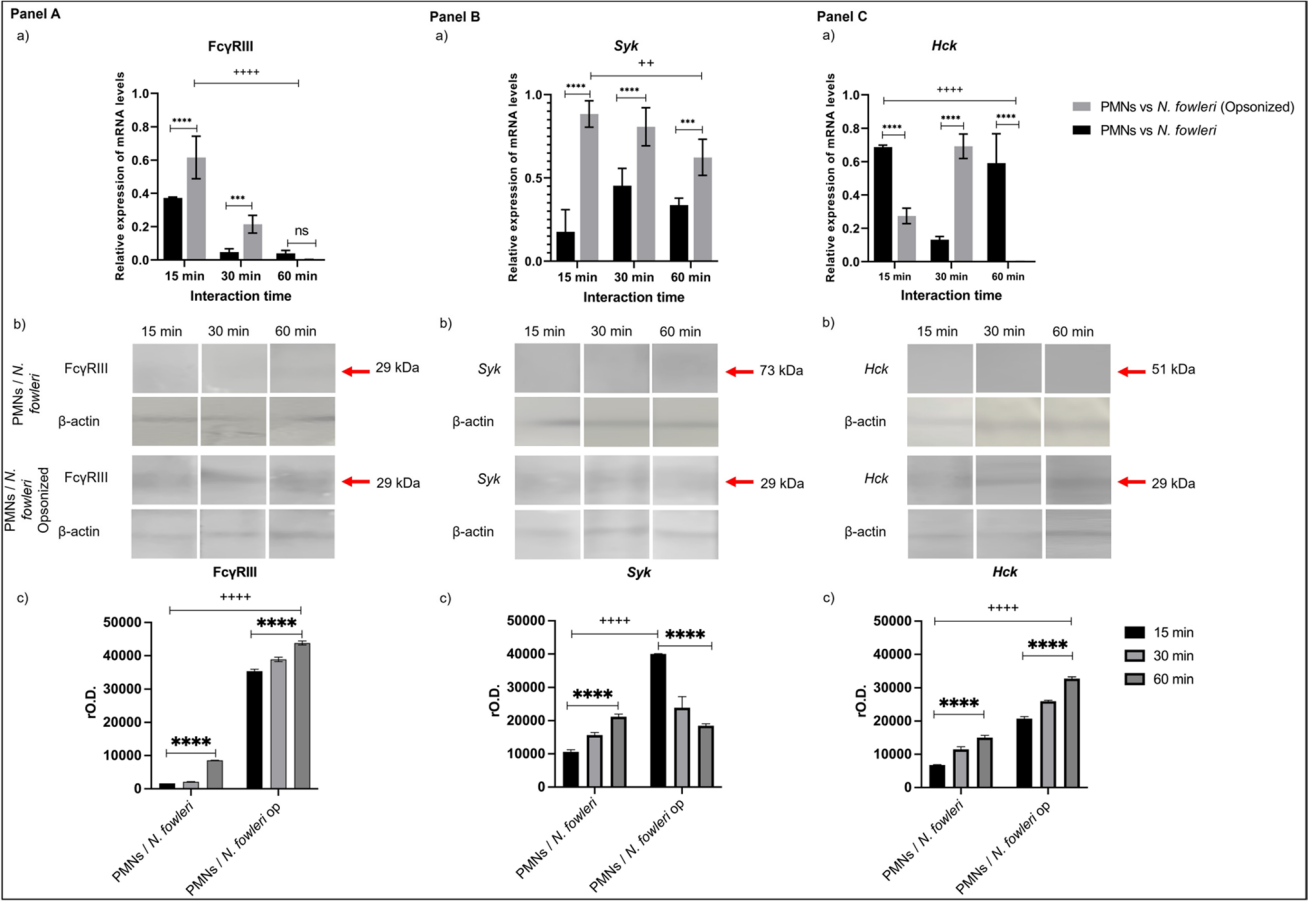
Expression of FcγRIII, *Syk*, and *Hck* in interacting PMNs. PMNs were purified from immunized and challenged mice and interacted with live *N. fowleri* trophozoites without opsonization or opsonized with IgG. The genes of **A** FcγRIII, **B** *Syk*, and **C** *Hck* were analyzed by RT-PCR and Western blot. The expression of the FcγRIII and *Syk* genes increased in interactions with opsonized amoeba compared to unopsonized amoeba; however, they decreased with increasing time. Likewise, protein expression is increased in PMNs interacting with opsonized amoeba, where at 60 min the maximum expression for FcγRIII and *Hck* is observed. The graphs show the mean ± SD of three independent experiments (*n* = 3). Two asterisks indicate significant difference of *P* < 0.01, three asterisks indicate significant difference of *P* < 0.001, four asterisks indicate significant difference of *P* < 0.0001 between interaction times, two plus signs indicate significant difference of *P* < 0.01, three plus signs indicate significant difference of *P* < 0.001, and four plus signs indicate significant difference of *P* < 0.0001 between the types of interaction

Like the previous results, analysis was performed for the FcγRIII, *Syk*, and *Hck* genes in PMNs of immunized and challenged mice that were interacted with opsonized or non-opsonized *N. fowleri* trophozoites (Fig. 6). The expression of the genes for FcγRIII is observed in the PMNs at 15 min of interaction with non-opsonized amoebas; however, the expression decreases as the interaction time increase. No significant differences are observed between 30 and 60 min of interaction (Fig. 6A, a). The FcγRIII mRNA levels were higher in PMNs interacted with opsonized *N. fowleri* compared with those PMNs interacted with non-opsonized amoebas., However, no significant differences were found at 60 min between interactions with opsonized and unopsonized *trophozoites* (Fig. 6A, a). Protein expression was also analyzed by Western blot; in the interactions with non-opsonized amoeba, no bands were observed at 15 and 30 min, in contrast to 60 min where a slight band with a rMW of 29 kDa was detected. Meanwhile, when PMNs were interacted with opsonized amoebas, the 29-kDa band is observed at 15, 30, and 60 min (Fig. 6A, b). The densitometric analysis of the Western blot showed that no significant differences are observed at 15 and 30 min, but a significant increase (*P* < 0.0001) at 60 min of interaction with PMNs with non-opsonized trophozoites was found (Fig. 6A, c). Similarly, in the interactions with PMNs and opsonized *N. fowleri*, no significant differences were observed at 15 and 30 min; however, a significant increment (*P* < 0.01) was observed at 60 min of interaction, which was higher (*P* < 0.0001) compared to the rest of the times and type of interaction (Fig. 6A, c).

At 15 min of interaction with non-opsonized trophozoites, *Syk* gene was expressed with no significant difference compared with the rest of the times (Fig. 6B, a). In contrast, the gene expression increases significantly (*P* < 0.0001) after interactions at 15, 30, and 60 min with opsonized amoebas compared to interactions with non-opsonized amoebas (Fig. 6B, a). However, no significant differences were observed between interaction times with *N. fowleri* opsonized. The *Syk* protein expression was observed in PMNs extract mainly at 60 min of interaction with non-opsonized amoebas. On the other hand, the expression of this protein was increased at the different times (15, 30, and 60 min) in PMNs that were interacted with opsonized trophozoites which was evidenced through the intensity of 73-kDa band. The densitometric analysis of the band reveals that there is no significant difference at 15 and 30 min of interaction with amoebas without opsonization, and a significant increase at 60 min is observed (*P* < 0.05) (Fig. 6B, b). At 15 min of interaction of PMNs with opsonized amoebas, the band was significantly higher (*P* < 0.0001), while it decreased as the time was increased.

The expression levels of the *Hck* genes were increased since 15 min of PMN interaction with *N. fowleri* without opsonization, and then these levels decreased at 30 min and augmented at 60 min; the Hck gene expression was similar at 15 and 60 min of interaction where significant differences were not shown. In contrast, when PMNs interact with opsonized amoeba at 15 min, the levels were low, but these increased significantly at 30 min (*P* < 0.0001) (Fig. 6C, a) and then decreased drastically after 60 min of interaction. Regarding HCK protein expression, we observed that PMNs interacted with non-opsonized trophozoites expressed faint bands corresponding to the molecular weight of 51 kDa, while, PMNs are interacted with opsonized amoeba, the bands were increased in all interaction times (Fig. 6C, b). The densitometric analysis showed that the levels of *Hck* of PMNs increase with the progress of the interaction time, where at 60 min the expression was significantly greater compared to the other interaction times (*P* < 0.0001) (Fig. 6C, c).

## Discussion

Antibody response has been associated in the protection against *N. fowleri* infection either in vitro or in vivo assays (Carrasco-Yepez et al. 2019; Rojas-Hernández et al. 2004b). In immunized mice with total extract of *N. fowleri* co-administered with CT and subsequently challenged with lethal doses of live trophozoites of *N. fowleri*, we obtained 100% of survival, as well as a higher production of specific IgA and IgG antibodies against the trophozoites in the lumen of the nasal mucosa (Carrasco-Yepez et al. 2014). In that work, we observed that antibodies bind to the surface of the trophozoites, immobilizing and thus preventing them from joining the apical side of the nasal epithelium, while PMNs that are surrounding the trophozoites also prevent the entry and infection of *N. fowleri* to the olfactory epithelium of the nasal cavity suggesting that one of the main protective mechanism against *N. fowleri* infection in previously immunized mice is the trophozoites phagocytosis given by PMNs cells through their receptors at the Fc fraction of antibodies (Carrasco-Yepez et al. 2010; Contis-Montes De Oca et al. 2016; Jarillo-Luna et al. 2008). These results led us to suggest that the Fc receptor participates in the mechanisms triggered for the protection.

The adaptive immune response through the production of specific IgG antibodies against pathogens is capable of activating PMNs by binding FcγRIII to antigen–antibody complexes, which leads to the activation of different effector mechanisms for the elimination of microorganisms (Huizinga et al. 1988; Wang & Jönsson 2019). When FcγRIII binds to the Fcs region of the antibodies, PMNs cross-link these receptors complex and initiate the activation of its effector functions through the phosphorylation of adapter proteins such as *Hck* and *Syk*, allowing the activation of nuclear factors that could produce cytokines, chemokines, or other molecules that participate in the destruction of the pathogens.

We found a large amount of IgG (IgG1, IgG2a, IgG3) in serum samples, nasal washes, olfactory epithelium, and in the lumen of the nasal cavity of immunized mice. It has been widely reported that the presence of serum IgG can mediate humoral responses in mucosal surfaces (Westerman et al. 2005), particularly, IgG1 that is the most abundant subclass in humoral response (Th2) (Mountford et al. 1994) and has the quality of responding to membrane proteins of pathogens like bacteria (Ferrante et al. 1990). In addition, the Th2 response has been shown to play a critical role in protection against *N. fowleri* as we have observed when STAT6-deficient mice are immunized with *N. fowleri* lysates plus Cry1Ac, the antibody response is minimal, compared to STAT6 + / + mice which, after immunizations and subsequent challenge with lethal dose of *N. fowleri*, had 100% survival (Carrasco-Yepez et al. 2010). The results of the present work agree with those obtained in our previous works where it was found high levels of IgG1, compared to the moderate levels of IgG2a (Carrasco-Yepez et al. 2014, 2010; Rojas-Hernández et al. 2004b). In addition, when mice were immunized with total extract of *N. fowleri* alone or co-administered with CT, an increment of the levels of IgG and IgA antibodies were detected in serum, nasal washes, and in the nasal cavity of mice. Furthermore, some important antigens of *N. fowleri* have been found highly immunogenic as the response of IgG and IgA is directed strongly towards some *N. fowleri* polypeptide bands (Carrasco-Yepez et al. 2013; Castillo-Ramírez et al. 2021; Cervantes-Sandoval et al. 2010; Gutiérrez-Sánchez et al. 2020).

On the other hand, there have been reports where CT inoculated by the intranasal route mainly induces Th2 responses, by an increase expression of IL-4, TGF-β, and IL-10, promoting the class-switch recombination in lymphocytes B and posterior differentiation of plasmablast to plasmatic cells (Lycke et al. 1990).

As we can observed in Fig. 1C–D, these results indicate that an effective Th2 response is being mounted against *N. fowleri* as IgG1 of serum and nasal washes from immunized mice recognized several antigens of *N. fowleri*; particularly some protein bands were recognized with a great intensity by this isotype. Regarding IgG2a and IgG3, the recognition of bands as well as their intensity was lower, particularly in nasal washes.

It is widely known that the production of the IgG2a is increased due to INFγ, a Th1 cytokine (Mosmann & Coffman 1989), which has been related to antibody cytotoxic responses (Jia et al. 2020). Additionally, IgG3 responds effectively against pathogen carbohydrates and binds to complement proteins, triggering the migration of inflammatory cells (Collins 2016; Perlmutter et al. 1978).

The response of the different subclasses of IgG can be found in the T-dependent and T-independent response (Deenick et al. 1999) against a wide variety of pathogens such as bacteria (Sidorin & Solov'eva 2011), viruses (Honda-Okubo et al. 2015), and protozoa (Carrasco-Yepez et al. 2014; Khomkhum et al. 2019; Ribeiro et al. 2018), which led to the recent postulation of the quartet model theory, suggesting that the co-expression of the subclasses of IgG achieves harmony instead of competition for an effective response to pathogens (Collins 2016), explaining the varied response obtained with the different specific IgG subclasses against *N. fowleri* that we found in our results.

Particularly, IgG1 and IgG2a would have activity blocking the amoeba, and consequently, the immunocomplexes (ameba-antibody) would be recognized mainly by the FcγRIII of PMN cells.

It has been widely described that IgG is the predominant antibody found in serum; meanwhile, low levels of IgG are exhibited in mucosal layers under homeostatic conditions (Macpherson et al. 2008); however, recently it has been proposed that the presence of IgG on mucosal surfaces may be actively involved in the prevention of the binding of pathogens to mucosal epithelial cells, as well as promoting the trapping of pathogens by mucus, neutralization, complement activation, and antibody-mediated cytotoxicity (Mascola et al. 2000; Robert-Guroff 2000). The fact that we found levels of IgG in nasal secretions and in nasal epithelium suggests that it could be arriving via the bloodstream by transudation since it has been reported that the macromolecules from the serum can exude through the tight junctions of epithelial cells (Scott et al. 1986; Serikov et al. 2002; Van Itallie & Anderson 2004). Studies have revealed that once the plasmablast leave the lymphoid nodes, they reach the lamina propria of the different mucosal membrane, where they differentiate into plasmatic cells due stimulation of IL-5 and IL-6 secreted by Th2 cells (Strober W, 2005). In addition, memory cells B and specific IgG antibodies have been proposed to accumulate in the lamina propria from inductor sites, and this same accumulation plays an important role in the protection against pathogens (VanCott et al. 1994).

It is interesting to note that the exudate process of PMNs from the lamina propria into the lumen, as we observed in Fig. 2, could be because the tight junctions between the epithelial cells are broken and that through these openings the PMNs gain access to the lumen, where PMNs would surround *N. fowleri* and later become activated by binding to the FcγRIII receptor. It has been observed that epithelial cells have high rates of cell division, to allow cell remodeling and homeostasis, among other functions, due to the presence of self-renewing stem cells (Blanpain et al. 2007; Moore & Lemischka 2006) which would allow the migration of molecules and cells such as PMNs. Also, it has been widely reported that tight and adherent junctions play an important role in determining the shape, function, and polarity, as well as the regulation of the permeability of epithelia (Baum & Georgiou 2011; Tsukita et al. 2001), which also react to stimuli, modulating cell division (Xiong et al. 2014); when these junctions are separated, the permeability increases greatly, inducing the activation of the immune system in the mucosal membranes (Suzuki 2013). Likewise, when epithelial renewal occurs, the tight junctions guide the reforming of the epithelial mechanical barrier (Le Bras & Le Borgne 2014). This could explain the pathways we found in the epithelium, since the epithelial cells of the olfactory epithelium, when sensing the presence of *N. fowleri*, could promote the formation of channels, allowing the activation of inflammatory cells and the exudation of these, as well as antibodies, obtaining an effective response against trophozoites. Carrasco-Yépez in 2014 also found that after immunizations, an effective immune response was obtained without damage to the epithelium, compared to that observed for infected mice, where tissue damage was intense as well as an exacerbated response against *N. fowleri* (Carrasco-Yepez et al. 2014).

Moreover Carrasco-Yépez et al. in 2019 observed that after the infection of mice with *N. fowleri* trophozoites, there was an accumulation of PMNs in the nasal cavity, which released extracellular traps trying to contain the advance of trophozoites; however, NETs appeared not to be effective in eliminating all amoebas (Carrasco-Yepez et al. 2019).

As it has been reported, antibodies carry out their effector functions due to the Fc receptors present in the immunologic cells (Horton & Vidarsson 2013; Takai 2005). Regarding to this, when we immunized and challenged the mice, we could observe detritus of *N. fowleri* accompanied by IgG and FcγRIII-positive cells, suggesting that the IgG antibodies arrived at the lumen either by transport or exudation while PMNs arrived by exudation. Consequently, *N. fowleri* is opsonized with IgG antibodies to form immunocomplexes to be recognized by the inflammatory cells which perform their effector functions resulting in the elimination of *N. fowleri*. It should be noted that this is the first time that the presence of FcγRIII in epithelial tissue of the nasal cavity of mice is reported, and its expression is also increased after the immunization scheme with *N. fowleri* lysates plus CT. This result reinforces the idea that epithelial cells play an important role in mediating the immune response against pathogens (Vroling et al. 2008). The expression of FcγRIII in epithelial cells of the nasal cavity in humans has recently been reported, when the stimulation of the receptors in the epithelial cells with IgG immunocomplexes (IgG-LPS) results in the release of IL-6 and IL-8 (Golebski et al. 2019).

It has been reported that regulation of Fc receptors may be mediated by antigen type (Leino & Lilius 1992), as seen in increased FcγRI on neutrophils in patients with Kawasaki disease (Nakatani et al. 2001), or decreased FcγRIII in HIV-1 patients (Boros et al. 1990). Unlike what was observed in control mice, Fig. 3, g–l shows the presence of *N. fowleri* in the nasal mucosa of immunized mice which triggers the exudation of many PMNs into the lumen of the nasal cavity as well as specific anti-*N. fowleri* antibodies production and an increase of the FcγRIII expression in PMNs and epithelial cells, leading the formation of immune complexes that bind and activate the FcγRIII in the apical and basal regions of the epithelium, suggesting an important role in the mucosal immunoprotection against *N. fowleri*.

PMNs not being in contact with antigens in bone marrow guarantee us that after purification, they are not preactivated to respond. Therefore, the response we obtained can be induced by the recognition and subsequent binding of PMNs to IgG-*N. fowleri* immune complexes.

In vitro assays showed that when PMNs were interacted with non-opsonized trophozoites of *N. fowleri,* the viability of the amoebas was slightly reduced. However, when PMNs were interacted with opsonized trophozoites, the number of viable trophozoites decreased dramatically. We suggest that the recognition of immunocomplexes by FcγRIII would increase in the destructive capacity of PMNs.

FcγRIII has ITAM motifs (Fodor et al. 2006), which when it is activated triggers signaling cascades to the nucleus for the cell to perform its effector functions. The first step is the activation of tyrosine kinases of the Src family that leads the activation of *Syk*, which subsequently phosphorylate ITAM tyrosines, and triggers multiple cellular functions (Bezman & Koretzky 2007). Therefore, we decided to evaluate the expression of genes of FcγRIII, *Hck*, and *Syk* in the nasal cavity of immunized mice and in vitro in interactions of PMNs with opsonized trophozoites by RT-PCR. Furthermore, we evaluated the protein expression of the same molecules by Western blot. The results showed that the expression of all the analyzed genes increases after immunizations in both epithelial cells and nasal passages. The presence of FcγRIII in epithelial cells of the nasal cavity and nasal passages suggests the involvement of these cells in the immune response against *N. fowleri*. Activation cascade kinases present in epithelial cells and nasal passages suggest that the epithelium is being activated by immunization which would trigger an efficient response against the challenge with *N. fowleri* trophozoites (Getahun & Cambier 2015; Golebski et al. 2019; Nimmerjahn & Ravetch 2008). It would be of great importance in the future to analyze the profile of cytokines secreted by epithelial cells in the protection model of PAM.

The activation of FcγRIII mediates among other responses the release of NETs, due to the activation pathways initiated by *Hck* and *Syk* (Futosi et al. 2013; Pecht 2018; Rosales 2007; Suzuki et al. 2000). The cascade triggers the phosphorylation of extracellular signal-regulated kinase (ERK), which also contributes to the activation of NADPH oxidase, followed by phosphorylation of the nuclear factor Elk-1, resulting in NETosis (Alemán et al. 2016; Behnen et al. 2014; García-García et al. 2009; Papayannopoulos et al. 2010). The relationship between mRNA expression and protein expression indicate that transcription occurs in periods less than 1 up to 10 min (Chen & Larson 2016; Chen et al. 2016). In addition, sudden and rapid changes may contribute to the dissolution of transcriptional condensates, which would provide negative feedback to stop transcription (Henninger et al. 2021; Sharp et al. 2021). This would explain the high levels that we have found in the first few minutes of interaction between PMNs and *N. fowleri* opsonized with IgG, and as time progresses, gene expression decreases. However, protein expression increases because of the translation of transcripted mRNAs in the early stages of cell activation (Desai et al. 2019), leading the expression of FcγRIII, *Hck*, and *Syk* by the interacted PMNs over the time.

In this paper, we suggest FcγRIII in PMNs and epithelial cells; furthermore, specific IgG subclasses against *N. fowleri* in the nasal mucosa have a more important role than previously thought. IgG opsonizes trophozoites forming immunocomplexes, which mediate the cellular response by binding the FcγRIII and triggering the activation of signaling cascades involving *Hck* and *Syk*, resulting in an orderly and effective immune response against *N. fowleri* trophozoites both in vivo and in vitro.

## Data Availability

All data generated or analyzed during this study are included in this manuscript.
